# GPS data from 2019 and 2020 campaigns in the Chesapeake Bay region towards quantifying vertical land motions

**DOI:** 10.1038/s41597-022-01864-8

**Published:** 2022-12-02

**Authors:** Gabrielle Troia, D. Sarah Stamps, R. Russell Lotspeich, James Duda, Kurt J. McCoy, William Moore, Philippe Hensel, Ryan Hippenstiel, Thomas McKenna, David Andreasen, Charles Geoghegan, Thomas P. Ulizio, Madeline Kronebusch, Joel Carr, David Walters, Neil Winn

**Affiliations:** 1 Virginia Tech, Blacksburg, VA USA; 2 U.S. Geological Survey, Virginia - West Virginia Water Science Center, Richmond, VA USA; 3 Hampton University, Hampton, VA USA; 4 National Geodetic Survey, Silver Spring, MD USA; 5 Delaware Geological Survey, Newark, DE USA; 6 Maryland Geological Survey, Baltimore, MD USA; 7 U.S. Geological Survey, Eastern Ecological Science Center, Laurel, MD USA; 8 National Park Service, Annapolis, MD USA

**Keywords:** Geophysics, Natural hazards

## Abstract

The Chesapeake Bay is a region along the eastern coast of the United States where sea-level rise is confounded with poorly resolved rates of land subsidence, thus new constraints on vertical land motions (VLM) in the region are warranted. In this paper, we provide a description of two campaign-style Global Positioning System (GPS) datasets, explain the methods used in data collection and validation, and present the experiment designed to quantify a new baseline of VLM in the Chesapeake Bay region of eastern North America. Data from GPS campaigns in 2019 and 2020 are presented as ASCII RINEX2.11 files and logsheets for each observation from the campaigns. Data were quality checked using the open-source program TEQC, resulting in average multipath 1 and 2 values of 0.68 and 0.57, respectively. All data are archived and publicly available for open access at the geodesy facility UNAVCO to abide by Findable, Accessible, Interoperable, Reusable (FAIR) data principles.

## Abstract (summary)

**Table:** 

Measurement(s)	motion of the Earth’s surface
Technology Type(s)	Global Positioning System
Sample Characteristic - Environment	Chesapeake Bay
Sample Characteristic - Location	United States

## Background

**Fig. 1:**
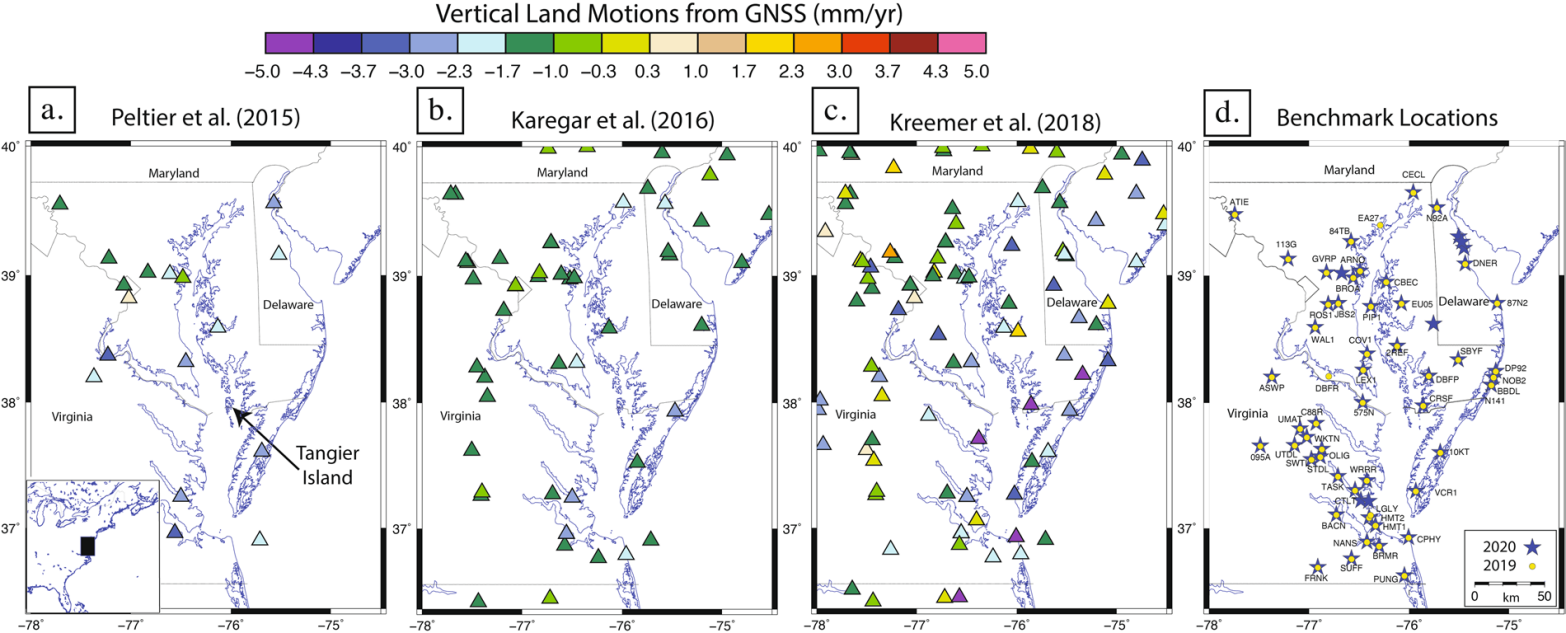
Vertical GPS velocity solution maps for the Chesapeake Bay region from previous studies illustrating the variability in solutions for the region. Tangier Island location is noted in (**a**). Benchmark sites are shown in (**d**). Yellow circles are sites from the 2019 campaign and blue stars are from the 2020 campaign. Base map coastlines are from the Global Self-consistent, Hierarchical, High-resolution Geography Database^27^.

The Chesapeake Bay, which is positioned along the eastern coast of North America, is a hotspot of land subsidence and relative sea-level rise^1,2^. In coastal regions, sea-level rise is of particular concern in areas where land subsidence occurs because relative sea-level rise is amplified by land subsidence. Potential drivers of vertical land motions (VLM) in the region include, but are not limited to, post-glacial rebound due to the retreat of the Laurentide Ice Sheet from the Last Glacial Maximum^3,4^, dynamic topography changes from mantle convection^5^, and aquifer compaction due to groundwater extraction^6,7^. Tide gauges may over or under-estimate the true rate of sea-level rise in areas experiencing VLM because local water level changes are confounded with VLM. The relative contributions of these two processes must be fully elucidated in order to improve coastal resilience efforts. One example of impact is that the inhabitants of Tangier Island (Fig. 1a) are estimated to have to relocate by the year 2065^8^.

VLM in the Chesapeake Bay region has been monitored using a variety of methods in recent decades, including leveling surveys^9^, borehole extensometer data^10^, GPS data^11^, and satellite interferometry^12^. Many measurements of VLM in the region have indicated subsidence, though at varying rates, and some studies indicate uplift in a few areas (Fig. 1a–c^11,13,14^), While the number of continuous GPS stations in the region has increased substantially since 2015, VLM measurements have varied. ^13^found an average regional subsidence rate of 1.93 mm/yr while^11^ and^14^ found average regional subsidence rates near 1.5 mm/yr. Moreover^13^, and^14^ suggest instances of uplift at varying rates and locations across the region. A recent study using InSAR-based measurements have also indicated subsidence rates as high as 6 mm/yr in some areas^15^. Thus, there is a need for a new baseline measurement of VLM in the Chesapeake Bay region, which we consider a quantification of VLM for a specific time period over a specific region.

The data described in this paper is part of a multi-year collaborative project funded by a grant from the U.S. Geological Survey (see Acknowledgements) that aims to constrain a new baseline measurement of VLM in the Chesapeake Bay region spanning a 5-year period that started in 2019. Two independent velocity solutions will be produced, using GAMIT-GLOBK and OPUS Projects to isolate long term and short term VLM signals and determine potential drivers. Outcomes of this project will provide a deeper understanding of geologic and anthropogenic processes in the area of study as well as aid coastal resilience efforts. Here we will describe the methods and data obtained during the first two (2019 and 2020) campaigns (Fig. 1d). A subsequent campaign occurred in 2021 and are two more are planned for 2022 and 2023.

## Methods

**Fig. 2:**
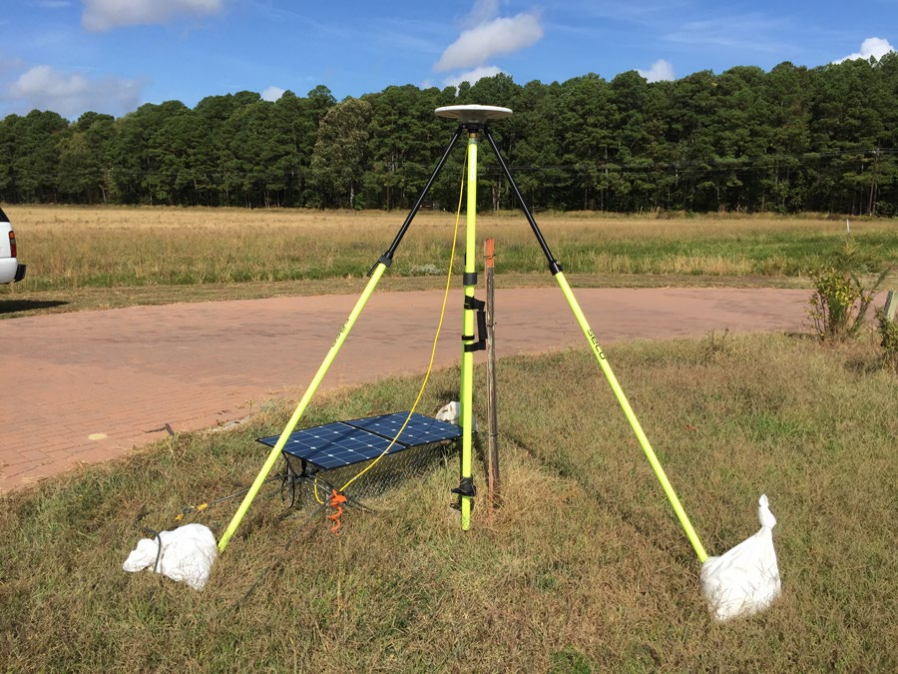
Example site 2REF with equipment fully set up during the 2019 campaign. Trimble Zephyr Geodetic 3 antenna and Trimble NetR9 receiver. Photo credit Philippe Hensel.

GPS campaigns were held in October of each year to minimize seasonal signals by observing in the same season. On average across both campaigns, observations at a given site yielded 108 hours of usable data, which is defined as at least 12 hours of continuous data collected on one day. Surveyors from collaborating agencies occupied 61 sites in 2019 and 57 sites in 2020 with marks of varying monumentation types, including deep rod, concrete monument, and rooftops. We planned to occupy the sites for at least 72 hours to obtain millimeter precision positions. An example of a site observed in this project, 2REF, is depicted in Fig. 2 with equipment fully deployed. Many of the benchmarks, including 2REF, are included in the National Geodetic Survey survey marks database^15^.

### Pre-survey

Before any campaigns were conducted, all sites were assigned unique 4-character identifiers (4CID’s). The 4CID’s were checked against several databases (UNAVCO^16^, Nevada Geodetic Laboratory^17^, International Global Navigation Satellite Systems Service^18^) to ensure they are unique identifiers. Due to the vast number of sites observed and the collaborative nature of this project, workshops were held in September prior to each campaign to ensure that surveying techniques such as equipment setup and takedown, tripod calibration, and tripod height measurement were performed consistently by all surveyors. For example, surveyors were instructed to orient their antennas to true north by correcting for magnetic declination, level their tripods, weight down each tripod, and fill out logsheets. Weekly virtual meetings were held leading up to each campaign to orchestrate coverage of all sites and review field methods.

### Survey

**Fig. 3:**
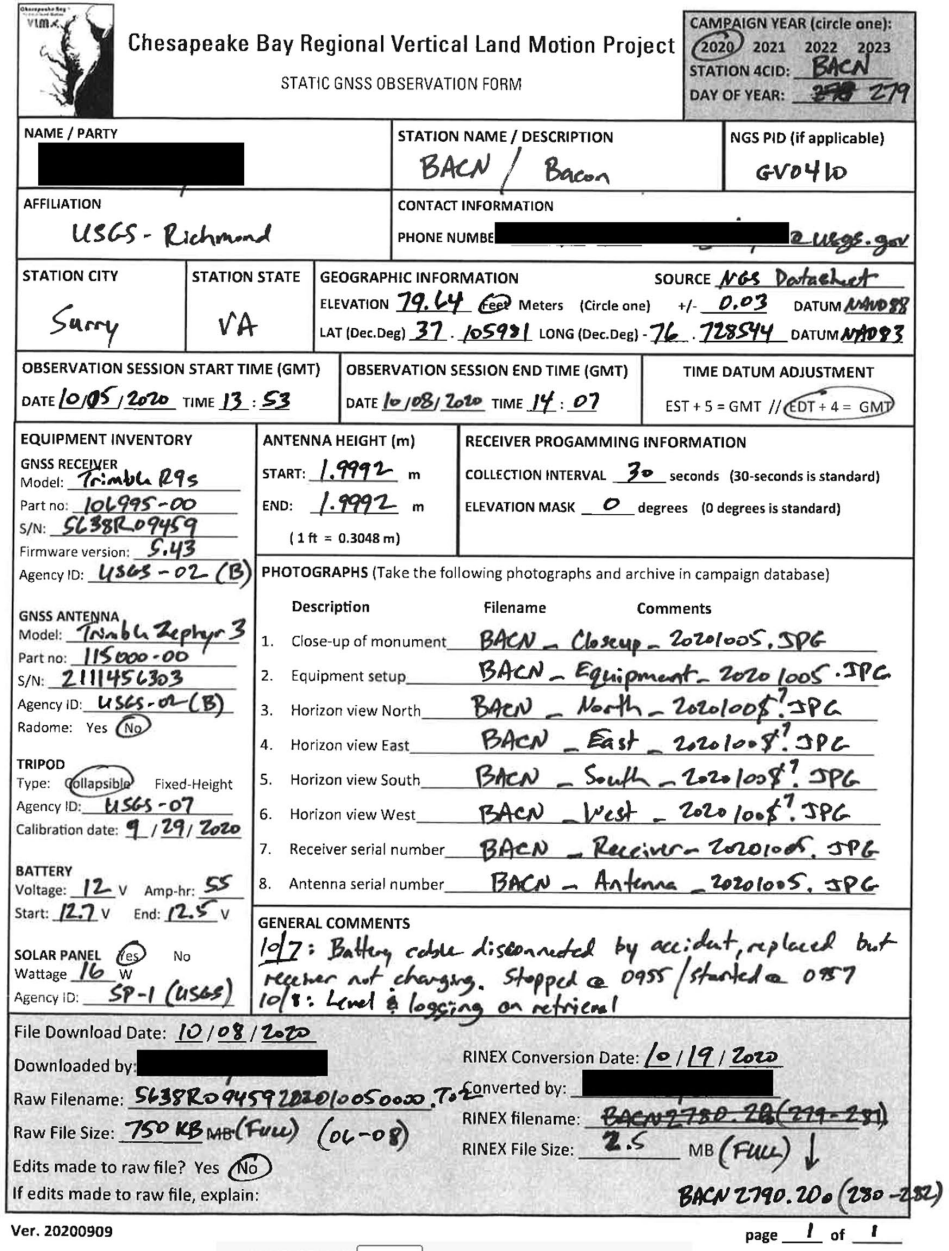
Example of a logsheet completed by surveyors during the 2020 campaign for site BACN.

Portable dual frequency Global Navigation Satellite System (GNSS) receivers were used to observe all sites, although a few sites were observed with GPS-only receivers. Despite GNSS capable receivers, we chose to archive only the GPS data. Trimble receivers NetR9 and Zephyr II antennas were the most common equipment; however a few integrated dual frequency receiver and antenna stations were used as well such as those manufactured by Ashtech, Javad, and CHC-X90. Station logsheets were created for surveyors to complete during the 2019 and 2020 campaign, which contained fields such as site location, monument description, and site ownership in addition to equipment specifications. Site photos were also included with these forms, indicating landmarks that can be used to help locate the site, as well as identify possible GPS signal obstructions. For each campaign, surveyors completed observation forms to record metadata specific to equipment and time of setup. These forms also contained notes on issues experienced during the campaign (e.g. receiver stopped working or equipment was damaged upon return). Figure 3 depicts an example of a completed observation form from the 2020 campaign. Surveyor name and agency, contact information, survey dates, mark designation, 4CID, receiver model and serial number, antenna type and serial number, antenna height, and photos of setup were the most critical metadata collected in order to perform a thorough validation of all data. Each institution involved (see co-author institutions and acknowledgments) coordinated their own team to manage the occupation of the network. The list of surveyors involved in the campaigns that are not coauthors are provided in the acknowledgments.

### Post-survey

**Fig. 4:**
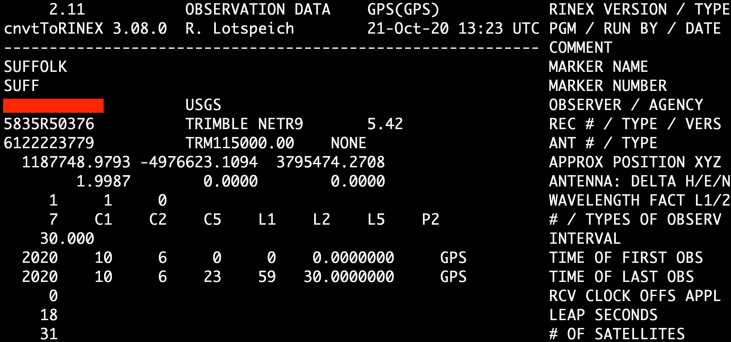
Example of a RINEX header completed with survey metadata with surveyor’s name marked out.

After the survey, we downloaded raw binary observation files from each receiver. Using runpkr00, TEQC, or other appropriate software, the raw files were converted to daily Receiver Independent Exchange (RINEX) 2.11 files and uploaded to a shared drive by each surveyor. The RINEX2.11 file version was used for both campaigns for consistency. RINEX2.11 files contain both GPS data and relevant metadata, making them easily transferable to future projects. The naming convention used is the standard format used by IGS and UNAVCO of “xxxxDOY0.Yyo,” where “xxxx” is the lowercase 4-character site identifier, “DOY” is the Day Of Year (001–366), “session”, “YY” represents the two-digit year of observation, and “o” denotes an observation file. Each RINEX file was reviewed and updated with accurate metadata in its header post-survey, including the surveyor’s name, agency responsible for surveying the station, equipment used, and antenna height that was recorded in the observation forms (Fig. 4). This data was cross referenced with photos taken by surveyors to confirm serial and model numbers and antenna heights.

## Data Records

The RINEX2.11 files discussed in this publication are archived for open access at UNAVCO^19,20^ and Zenodo^21,22^ to abide by Findable, Accessible, Interoperable, Reusable (FAIR) data principles^23^. Logsheets for each station are also archived with each dataset; PDF files are named according to the station and date of the survey. Logsheets from the 2019 campaign contain additional information (e.g. detailed site descriptions) that would be helpful for anyone who wishes to locate and observe any sites in the future, although the 2020 campaign logsheets also include enough information for future surveyors to locate the sites.

## Technical Validation

**Fig. 5:**
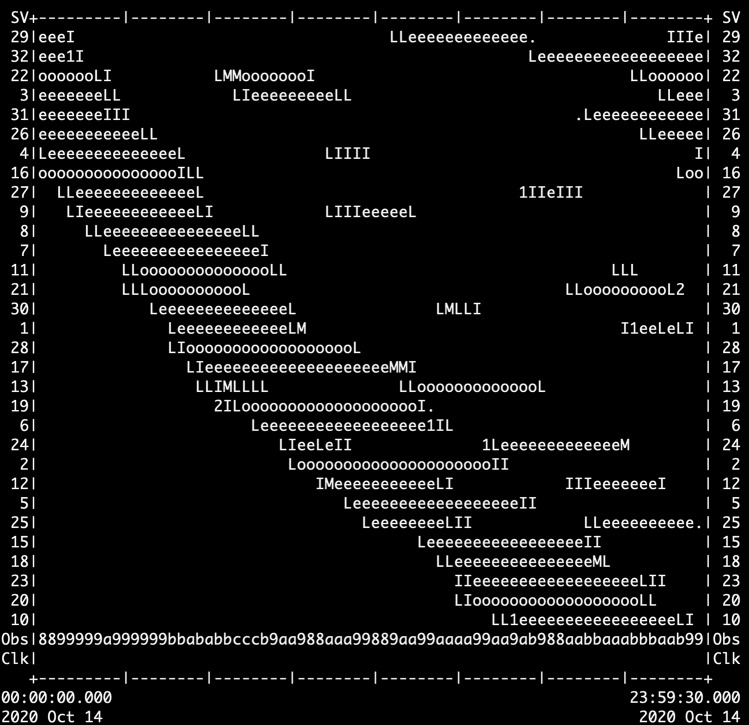
Example of an ASCII time plot from a summary file of a typical high quality RINEX2.11 file.

**Fig. 6:**
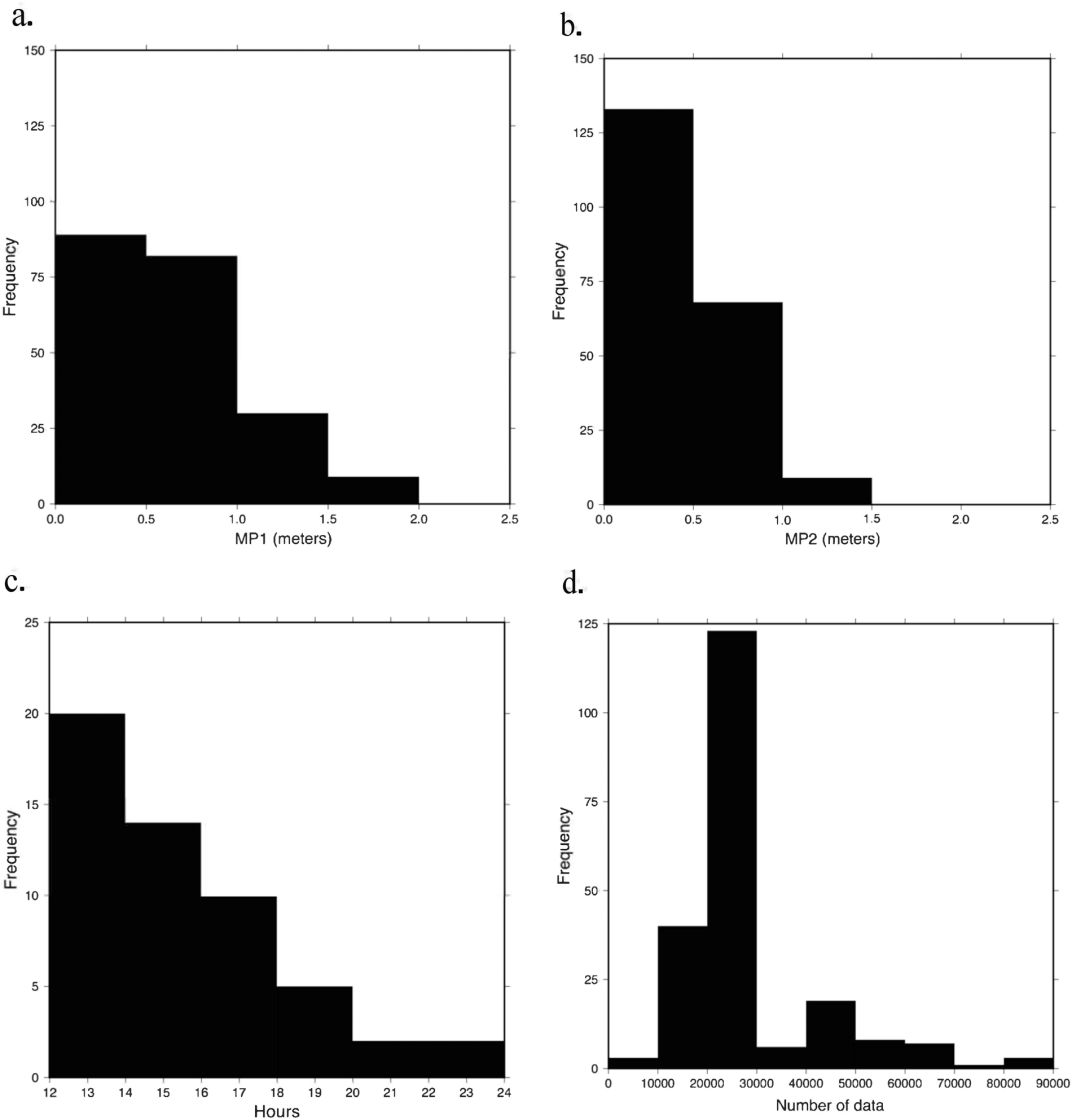
Quality check statistics as histograms for usable 2019 RINEX files. The frequency of the number of days of year is plotted against the quality check statistics. (**a**) Multipath 1, (**b**) Multipath 2, (**c**) Number of observations per file, (**d**) Hours of data per file.

**Fig. 7:**
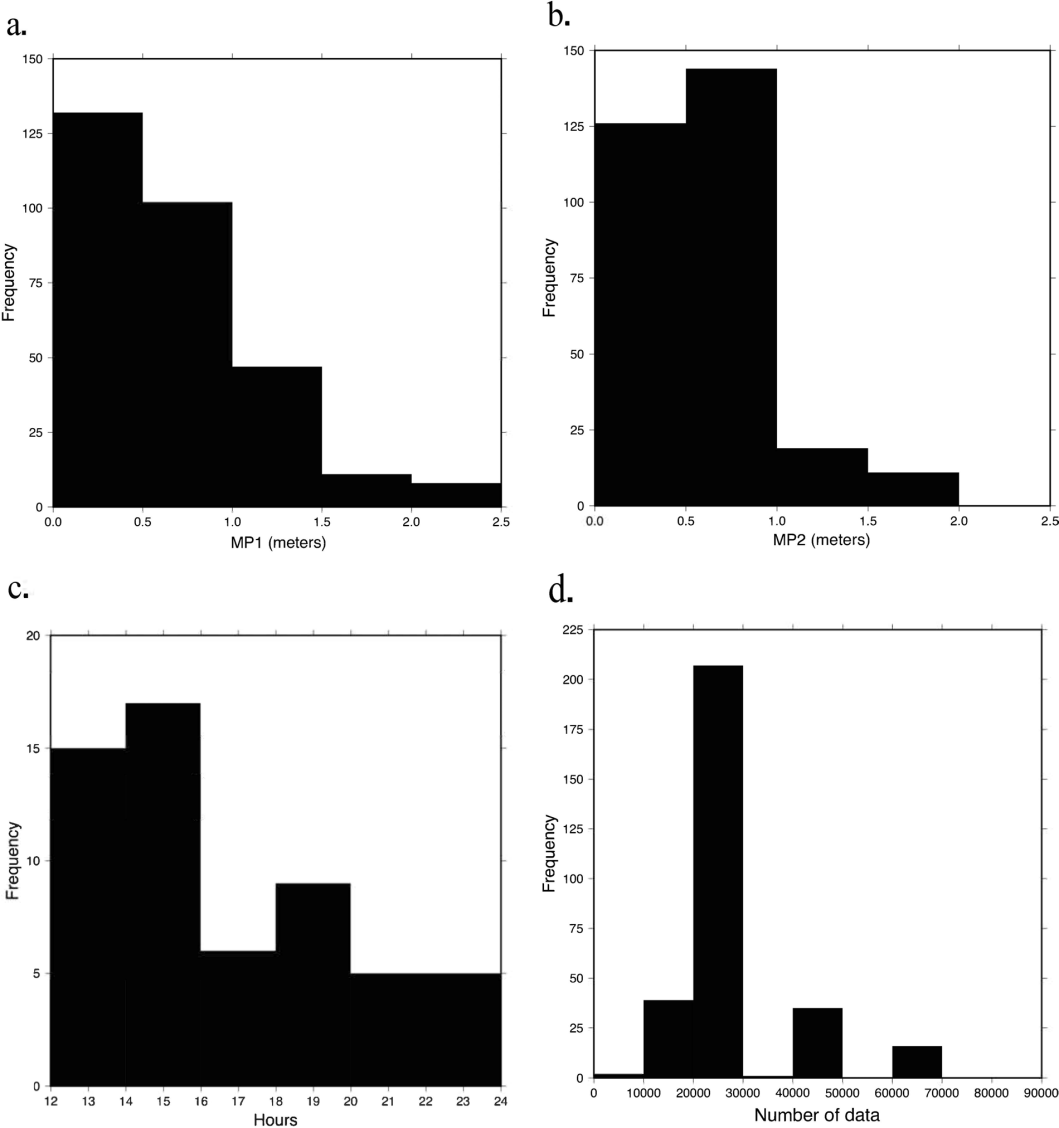
Quality check statistics as histograms for usable 2020 RINEX files. The frequency of the number of days of year is plotted against the quality check statistics (**a**) Multipath 1, (**b**) Multipath 2, (**c**) Number of observations per file, (**d**) Hours of data per file.

Using the open-access program TEQC^24^, a summary file was generated (.yyS) for each daily RINEX2.11 file to assess data quality (note that TEQC is no longer being supported by its developer, and the source code is archived at UNAVCO^25^). Quality check statistics are visually represented in the ASCII time plot present in each summary file (Fig. 5) where each number on the sides are satellites, e and o indicate key signals were observed, L indicates loss of lock with the satellite, M indicates both multipath signals slipped, and I indicates an ionospheric delay slip occurred. Multipath values, the number of observations per daily file, and hours of data were used as metrics to assess the quality of the data collected in each campaign. The multipath value indicates the distance delay (in meters) of a signal between a satellite and receiver due to reflections of the signal off of physical obstructions such as trees or buildings for the L1 (MP1) and L2 (MP2) frequencies^20^. Figures 6 and 7 present multipath (MP1 and MP2) values (Figs. 6a,b,7a,b), hours of data per daily RINEX file (Fig. 6c,7c), and number of data points collected per daily file (Figs. 6d,7d). RINEX files with less than approximately 12 hours of data and sites with less than 24 hours of data total were excluded from each dataset and not archived at UNAVCO. The following statistics that are presented were obtained only from the archived datasets.

### 2019 Campaign data

A total of 604 Mb of usable data were obtained during the 2019 campaign. Usable data means at least 12 hours of continuous data were collected. Usable data from the 2019 campaign spanned day of year (DOY) 278 to 294 (5–21 October). Days on which the greatest number of receivers collected data were DOY 281, 282, and 283 (8, 9, 10 October), with 44 being the greatest number of receivers running on the same day (DOY 283). Due to the complex logistics of the project, the least number of receivers recording was 1 on DOY 278, 279 (5 and 6 October). The average MP1 and MP2 values for the 2019 campaign data are 0.64 and 0.49 m, with standard deviations of 0.38 and 0.22 m (Fig. 6a,b). Except for DOY 294, at least one GPS station recording on a particular day collected 24 hours of data. The range of hours collected per daily RINEX file was 12.09 to 24, and the average hours of data was 21.9 (Fig. 6c). For the entire campaign 2019, the number of observations collected per daily RINEX file on average was 28,640 (Fig. 6d). The 2019 campaign resulted in the archival of data from 52 stations that collected usable data with an average of 87.87 hours of data per station.

### 2020 Campaign data

A total of 827 Mb of usable data was collected during the 2020 campaign. Usable data from the 2020 campaign spanned DOY 279 to 303 (5–29 October). The greatest number of receivers collected usable data on DOY 288 and 289 (14 and 15 October), with 28 receivers being the greatest number of receivers to collect usable data on the same day (DOY 289). 1 receiver collecting on any given day was the lowest, which occurred on DOY 298–300 (24–26 October) due to the complex logistics of the campaign. The average MP1 and MP2 values for the 2020 campaign were 0.71 and 0.62 m, with standard deviations of 0.44 and 0.31 m (Fig. 7a,b). On average, each usable RINEX2.11 file contains 22.56 hours of data with a range of 11.9 to 24 hours of data (Fig. 7c). The average number of data collected per daily RINEX2.11 file was 29,129 (Fig. 7d). The 2020 campaign resulted in the archival of data from 54 stations with an average of 118.99 hours of usable data per station, which is a 42% increase from the previous year. There were 3 stations that did not obtain sufficient data to be archived. Although multipath values are higher in the 2020 data, the significant increase in hours of usable data may ultimately provide more precise positions after processing.

## Data Availability

The data provided in this paper was processed on a desktop computer running Scientific RedHat Linux 6 in the tcsh shell. The open-source conversion programs runpkr00 (available at UNAVCO via Trimble, www.unavco.org) and TEQC (also available at UNAVCO) were used to convert native receiver file formats to RINEX2.11 files when RINEX2.11 files were not available from surveyors. Vim, an open-source visual editor, was used to edit headers of RINEX2.11 files and to view summary files created during quality checking. Data quality checking was also completed using TEQC. Technical figures in this paper were created using the open-source program Generic Mapping Tools (GMT^26^).

## References

[1] Sallenger AH, Doran KS, Howd PA (2012). Hotspot of accelerated sea-level rise on the Atlantic coast of North America. Nature Climate Change.

[2] Eggleston, J. & Pope, J. P. Land subsidence and relative sea-level rise in the southern Chesapeake Bay region. US Department of the Interior, US Geological Survey (2012).

[3] Davis JL, Mitrovica JX (1996). Glacial isostatic adjustment and the anomalous tide gauge record of eastern North America. Nature.

[4] Karegar MA, Dixon TH, Engelhart SE (2016). Subsidence along the Atlantic Coast of North America: Insights from GPS and late Holocene relative sea level data. Geophysical Research Letters.

[5] Boschi, L., Faccenna, C., & Becker, T. W. Mantle structure and dynamic topography in the Mediterranean Basin. Geophysical Research Letters, 37(20) (2010).

[6] DePaul VT, Rice DE, Zapecza OS (2008). Water‐level changes in aquifers of the Atlantic coastal plain, predevelopment to 2000, U.S. Geol. Surv. Sci. Invest. Rep..

[7] Boon JD, Brubaker JM, Forrest DR (2010). Chesapeake Bay land subsidence and sea level change App. Mar. Sci. and Ocean Eng., Rep..

[8] Schulte DM, Dridge KM, Hudgins MH (2015). Climate change and the evolution and fate of the Tangier Islands of Chesapeake Bay, USA. Scientific reports.

[9] Holdahl SR, Morrison NL (1974). Regional investigations of vertical crustal movements in the US, using precise relevelings and mareograph data. Tectonophysics.

[10] Pope JP, Burbey TJ (2004). Multiple‐aquifer characterization from single borehole extensometer records. Groundwater.

[11] Sella, G. F. *et al*. Observation of glacial isostatic adjustment in “stable” North America with GPS. Geophysical Research Letters, 34(2) (2007).

[12] Buzzanga B, Bekaert DP, Hamlington BD, Sangha SS (2020). Toward Sustained Monitoring of Subsidence at the Coast Using InSAR and GPS: An Application in Hampton Roads, Virginia. Geophysical Research Letters.

[13] Peltier WR, Argus DF, Drummond R (2015). Space geodesy constrains ice age terminal deglaciation: The global ICE‐6G_C (VM5a) model. Journal of Geophysical Research: Solid Earth.

[14] Kreemer C, Hammond WC, Blewitt G (2018). A robust estimation of the 3‐D intraplate deformation of the North American plate from GPS. Journal of Geophysical Research: Solid Earth.

[15] *National Geodetic Survey. Survey Marks Database* https://geodesy.noaa.gov/datasheets/ (2022).

[16] *UNAVCO. GNSS/GPS Dataset DOIs* https://www.unavco.org/data/doi/search/search.html (2022).

[17] *Nevada Geodetic Laboratory. List of All GPS Stations Processed by NGL* http://geodesy.unr.edu/NGLStationPages/GlobalStationList (2022).

[18] International Global Navigation Satellite System Service. Network, https://igs.org/network/#station-map-list (2022).

[19] Troia G (2020). UNAVCO, Inc.

[20] Troia G (2022). UNAVCO, Inc.

[21] Troia G (2020). Zenodo.

[22] Troia G (2020). Zenodo.

[23] Wilkinson MD (2016). Principles for scientific data management and stewardship. Scientific data.

[24] Estey, L., & Wier, S. Teqc Tutorial: basics of Teqc use and Teqc products. UNAVCO Inc.: Colorado, CO, USA (2014).

[25] TEQC. UNAVCO. (n.d.), https://www.unavco.org/software/data-processing/teqc/teqc.html (2022).

[26] Wessel P, Smith WHF, Scharroo R, Luis J, Wobbe F (2013). Generic Mapping Tools: Improved Version Released, EOS Trans. AGU.

[27] Wessel, P., & Smith W. H. F. A Global Self-consistent, Hierarchical, High-resolution Geography Database (GSHHG), Version 2.3.7, https://www.soest.hawaii.edu/pwessel/gshhg/ (2017).

